# Subcellular electrical stimulation of neurons enhances the myelination of axons by oligodendrocytes

**DOI:** 10.1371/journal.pone.0179642

**Published:** 2017-07-03

**Authors:** Hae Ung Lee, Agata Blasiak, Devansh R. Agrawal, Daniel Teh Boon Loong, Nitish V. Thakor, Angelo H. All, John S. Ho, In Hong Yang

**Affiliations:** 1 Singapore Institute for Neurotechnology, National University of Singapore, Singapore, Singapore; 2 Department of Biomedical Engineering, School of Medicine, Johns Hopkins University, Baltimore, Maryland, United States of America; 3 Department of Neurology, Johns Hopkins School of Medicine, Baltimore, Maryland, United States of America; 4 Department of Electrical and Computer Engineering, National University of Singapore, Singapore, Singapore; Instituto Cajal-CSIC, SPAIN

## Abstract

Myelin formation has been identified as a modulator of neural plasticity. New tools are required to investigate the mechanisms by which environmental inputs and neural activity regulate myelination patterns. In this study, we demonstrate a microfluidic compartmentalized culture system with integrated electrical stimulation capabilities that can induce neural activity by whole cell and focal stimulation. A set of electric field simulations was performed to confirm spatial restriction of the electrical input in the compartmentalized culture system. We further demonstrate that electrode localization is a key consideration for generating uniform the stimulation of neuron and oligodendrocytes within the compartments. Using three configurations of the electrodes we tested the effects of subcellular activation of neural activity on distal axon myelination with oligodendrocytes. We further investigated if oligodendrocytes have to be exposed to the electrical field to induce axon myelination. An isolated stimulation of cell bodies and proximal axons had the same effect as an isolated stimulation of distal axons co-cultured with oligodendrocytes, and the two modes had a non-different result than whole cell stimulation. Our platform enabled the demonstration that electrical stimulation enhances oligodendrocyte maturation and myelin formation independent of the input localization and oligodendrocyte exposure to the electrical field.

## Introduction

Myelin sheaths play pivotal roles as an axon isolator and a conductor of electrical signals in nerve system. In the recent years, the function of myelination has been identified to not only potentiate conduction of neural signals but to affect neural function and plasticity as well (reviewed in [1, 2]). The *in vivo* evidences of such myelin-based plasticity mechanisms were demonstrated in behavioral studies in both juvenile and adult mice. Social isolation of two-week-old pups resulted in thinner and lower number of myelin sheaths, which in turn affected social interactions and working memory [3]. Similarly, socially isolated adult mice demonstrated thinner myelin sheaths, lower level of gene expression specific to myelination, and behavioral deficits. The two latter effects were reversed in 4 weeks after reintroducing social stimuli, emphasizing the remodeling ability of myelination process [4]. Finally, the generation of myelin-forming oligodendrocyte (OLs) was shown to correlate with motor learning—teaching a mouse a complex running task increased OLs formation, while a knockout animal with reduced oligodendrogenesis demonstrated impaired learning performance [5]. Those observations are indicative of a link between environmental stimuli, central nervous system (CNS) plasticity and myelination.

On a cellular level, axon conductivity can be dynamically affected by the changes in a structure of myelin sheath, including modulation of its thickness, length, and axon coverage [2, 6–8]. In turn, it has been shown that axon activity itself, although not prerequisite for OLs wrapping process [9, 10], supports oligodendrogenesis [11], induces *de novo* myelin formation [12, 13] and increases the thickness of the sheaths [14]. As the environmental stimuli are converted into neuronal circuit activity patterns, the neural activity-dependent myelination may play a crucial role in myelin-based regulation of CNS cognitive activity and learning. Those mechanisms remain unclear.

A common system for investigating myelination mechanisms is mixed neuron/glia co-culture in a dish or multi-well plate. Although easy to manipulate, these systems hinder identification of the mediator for signaling molecules, whether oligodendrocytes or neurons. Furthermore, they do not recapitulate the *in vivo* scenario, where neuronal cell bodies and majority of myelinating axons in a brain are spatially concentrated in different areas. Compartmentalized co-culture systems have been used to separate neuronal cell bodies in space and milieu, facilitating exclusive interaction of oligodendrocytes with distal axons [15, 16]. These models enable dissection of subcellular myelination mechanisms, which are important considering a focal scope of neuron activity stimulation with e.g., optogenetic technique [17] or electrodes. Compartmentalized platforms have been successfully integrated with electrical stimulation for studying activity dependent myelination [13, 18, 19]. However, these studies did not allow exploring if the increased myelination is driven solemnly by electrical stimulation of neurons, or does oligodendrocyte stimulation contribute to it too.

In this study, we describe a compartmentalized platform for whole cell and subcellular electrical stimulation of neurons, cell bodies and axons, with and without simultaneous stimulation of oligodendrocytes. We performed a set of simulations to understand the characteristics of the electric field and ensure restricted stimulation input. The simulations further demonstrate the importance of the compartment sizes and electrode positions within them. Finally, we show that subcellular stimulation enhances distal axon myelination on the same level as whole cell stimulation independent whether oligodendrocytes exhibited the electrical field or not.

## Material and methods

### Microfluidic platform

The fabrication process of the two-chamber microfluidic platforms connected by a set of parallel microchannels (10-μm-wide, 2.5-μm-high and 500-μm-long) followed a previously published protocol [19–21]. Briefly, the masters were fabricated by standard photolithography, and negative photoresist SU8 was used as the master layer for silicon (Si) wafer. The devices were prepared in polydimethylsiloxane (PDMS, Dow Corning Sylgard 184 Silicone elastomer) by mixing a base with a curing agent in 10:1 ratio, pouring on the Si wafer, degassing it in a vacuum for 1hr and curing at 70°C for 2 hours. After curing, the chambers were punched out with rectangular shaped punches prepared by reshaping circular Ø 8 mm biopsy punches (Ted Pella). The PDMS pads were bonded to glass coverslips (thickness 1, Menzel Glaser) by oxygen plasma treatment (Femto Science) and were sterilized by autoclaving (120°C for 30 min).

### Electric field simulations

**Fig 1:**
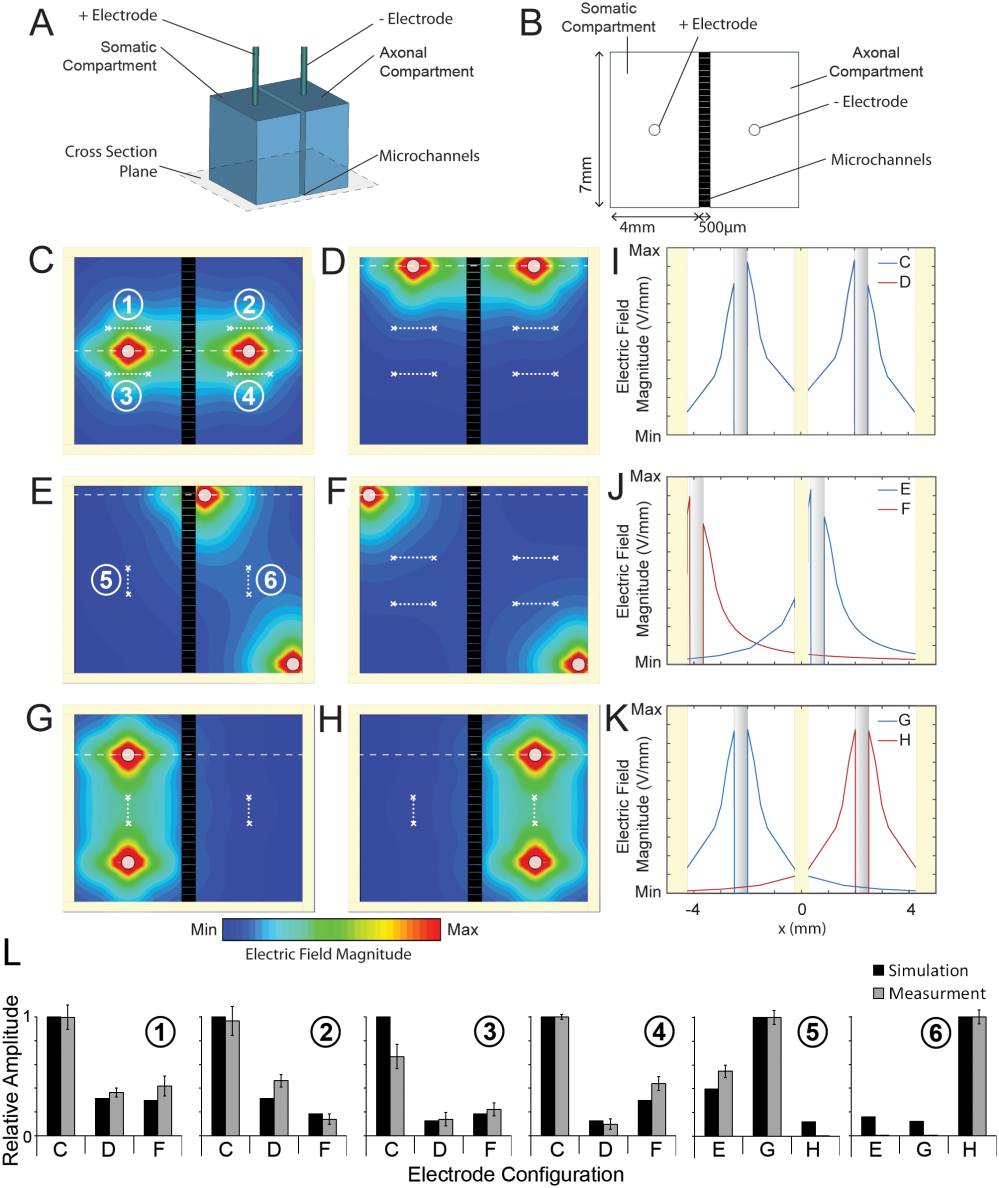
Electric field amplitude within the compartments. (A) Schematic of the simulation model. (B) Cross-section of model along indicated plane. (C-H) Contour map of electric field amplitude within compartments for different stimulation electrode configurations. (I-K) Profile of electric field amplitude along dashed lines in (C-H) spanning two wells. (L) Measured (gray; relative electrical potential difference) and simulated (black; relative electric magnetic field) at 6 locations, as numbered in (*C*) and (*E*) and outlined (white x and dotted lines) in all electrodes configurations (*C-H*). The data for G and H come from the same set of simulations and measurements. Measurement data are mean ± S.D. For individual data points see S2 Fig.

**Fig 2:**
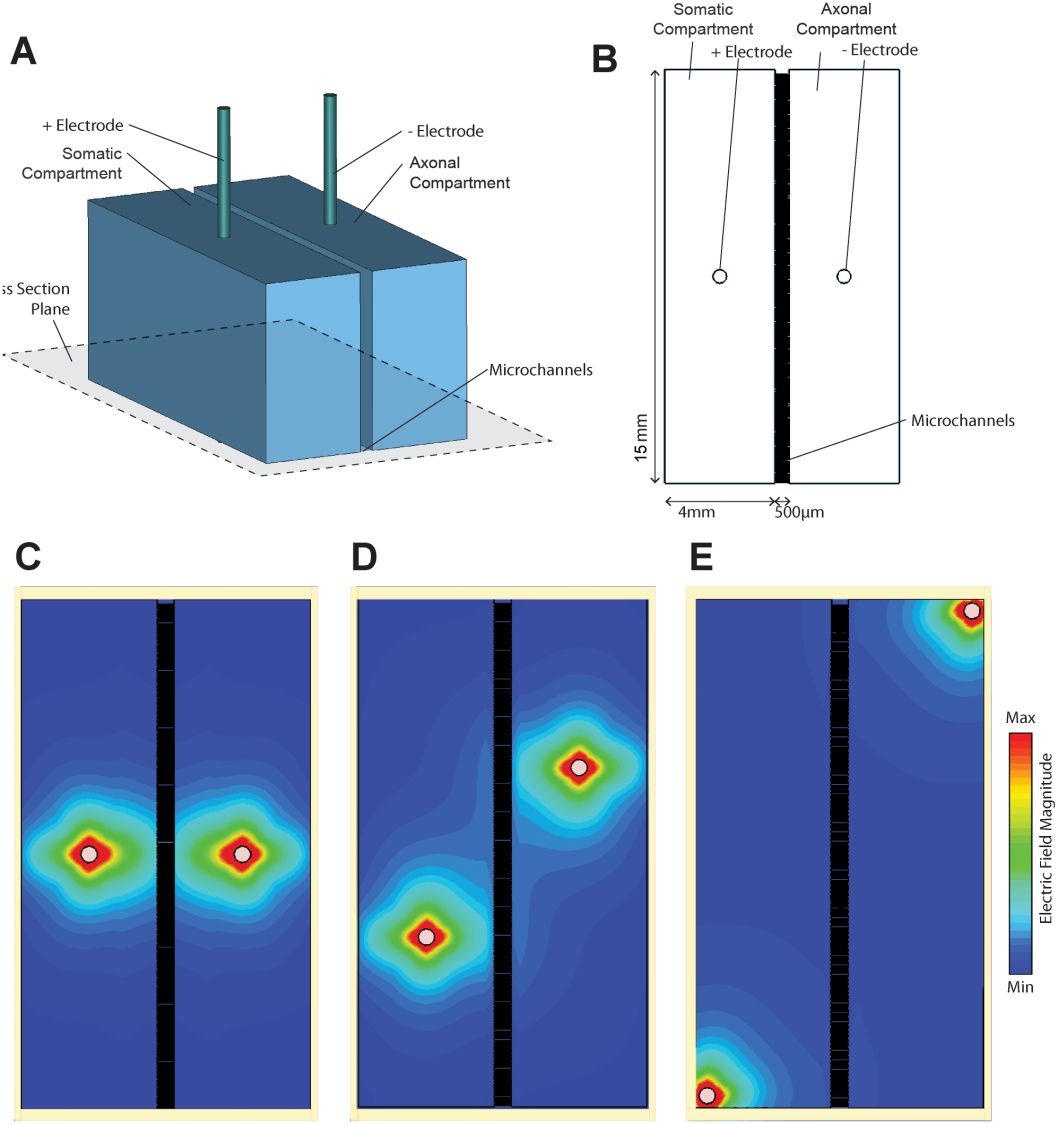
Electric field strength within the long compartments. (A) Schematic of the simulation model with larger compartment length (15 mm). (B) Cross-section of model along indicated plane. (C-E) Contour maps of electric field magnitudes within the compartments for different electrode configurations.

Electric field simulations were performed using a commercial finite-element electrostatic solver (CST Computer Simulation Technology). Electrodes were modelled using Pt cylinders of 0.25 mm radius. Simulations for the custom microfluidic system placed the electrodes in 2 extreme and 1 central positions within the 7 mm long × 4 mm wide compartments (connected by 500 μm long microchannels) to model whole cell and focal stimulation (Fig 1). Simulations for the commercial compartmentalized system also used 2 central and 1 extreme configuration for the electrodes within the 15 mm long × 4 mm wide compartments connected by 500 μm long microchannels (Fig 2). The media in the compartments were set to saline (relative dielectric permittivity εr = 80) [22, 23] and the surrounding material to PDMS (εr = 3)[24]. The potential across each pair of electrodes was set to 3 V as used in experiments, and the field solved using open boundary conditions. Following simulations, the field magnitude was computed using MATLAB (Mathworks) in order to represent the selectivity of the stimulation under worst-case orientation of individual axons relative to the field direction.

### Cell culture

This study was carried out in strict accordance with the recommendations by the Guide on Responsible Care and Use of Laboratory Animals by the National University of Singapore. The protocol was approved by the NUS Institutional Animal Care and Use Committee (Protocol Number: R14-1635). All efforts were made to minimize animal suffering. Dorsal root ganglion (DRG) neurons were collected from E13 ICR mice or E15 SD rat embryos as described previously [18]. The animal was sacrificed by CO_2_ asphyxiation. Dissected DRGs were digested by 20 min long incubation in 0.125% trypsin-EDTA (Life Technologies) at 37°C, followed by trituration. The sterile PDMS devices were coated with 0.1 mg/ml poly-D-lysine (PDL) and 5 μg/mL Laminin (Life Technologies) by overnight incubation in 4°C. Dissociated cells (approximately 160,000 cells/cm^2^) were seeded in the somatic chamber of the devices in Neurobasal medium (Gibco) supplemented with 1% Penicillin / Streptomycin (P/S, Gibco), B27 (Gibco), GlutaMAX-1 (Life Technologies), and nerve growth factor (NGF, 20 ng/mL; R&D systems). The cultures were maintained *via* half-volume media changes every 2–3 days. Flurodeoxyuridin (FudR, 13 μg/mL; Sigma) and Uridine (33 μg/mL; Sigma) were added for 4–6 days to eliminate non-neuronal populations.

Oligodendocyte precursor cells (OPCs) were prepared from the brains of postnatal day 1 mice pups. The pups were sacrificed by decapitation. The brain was dissected to isolate the two cortices. The meninges of the cortices were carefully removed and the cortices were minced into small pieces before dissociation with Neural Tissue Dissociation Kits (P) (Miltenyi Biotec) followed by processing with gentleMACS^™^ Dissociator (Miltenyi Biotec). Dissociated brain cells were tagged with anti-O4 microbeads (Miltenyi Biotec) and O4-positive OPCs were purified by magnetic-activated cell sorting (MACS Separator; Miltenyi Biotec). The purity of separated OPCs was verified by anti-O4-PE (Miltenyi Biotec), and was found to be 85–90%. Newly purified OPCs were plated (100,000 cells/cm^2^) in an axonal chamber of 10 day old mice DRG culture and demonstrated good viability (S1 Fig). DMEM containing Holo-transferrin (49.5 mg/L; Sigma), Bovine insulin (5 mg/L), progesterone (0.06 mg/L), putrescine (16.1mg/L), selenite (0.05 mg/L)], and 0.5% fetal bovine serum (FBS) were used to maintain DRG/OPCs co-cultures. Co-cultures were maintained in the absence of added growth factors for a total of 14 days (10 day old DRGs are NGF-independent) with half-volume media changes every 2–3 days.

### Electrical stimulation

Neuronal activity was induced using 0.5 ms biphasic pulses at 10 Hz. Pulse trains were applied for 0.5 s every 2 s. The platinum electrodes that extended down into the platform were connected to a constant voltage power supply of 3 V. The stimulation pulses were applied for 1 h daily using a MultiStim System D-330 stimulator (Digitimer) [13, 18, 19, 25]. The cultures remained in the incubator (37°C, 5% CO_2_) during the stimulation. The platforms containing electrodes that were not connected to the power source served as unstimulated controls.

### Electric potential measurements

Electric potentials were measured with 30-gauge silver-plated copper wires (Farnell) connected to mixed domain oscilloscope (Tektronix MDO3012). Stimulation electrodes were first positioned in a 2-mm thick PDMS pad for stabilization. Larger electrodes were placed by punching Ø 1mm holes using a biopsy puncher (Ted Pella). Thin measurement electrodes were placed using a 22.1 gauge needle (BD Sciences) to prick the PDMS pad. Stimulation pulses of 50 V were applied using the same equipment as for the ESTIM of the cells; the average height of 6–9 peaks was measured and normalized to the maximum value observed for each paradigm: whole cell or focal stimulation. The measurements for each configuration were performed by changing the positions of the stimulation electrodes without altering the measurement electrodes. Separate pads were used for whole cell (Fig 1C, 1D and 1F) and focal (Fig 1E, 1G and 1H) stimulation paradigms.

### Calcium imaging and analysis

Calcium imaging and analysis were performed as published before [26]. Briefly, at 5–7 DIV rat DRG neurons cultured in microfluidic devices were incubated with 1.4 μM of Fluo4 AM calcium dye (Invitrogen) for 40 min at 37°C. After dye loading, 90 μL out of 100 μL was replaced with the imaging medium (Neurobasal medium without phenol red (Gibco) supplemented with GlutaMAX-1, B27 and 20 ng/mL NGF) and incubated for 20 min in 37°C for a recovery. Image analysis was performed with FiJi software. The background of all images was subtracted using the “rolling-ball” background subtraction. 10 DRG cell bodies with detectable activity were selected and the region of interest (ROI) was set to the parameters of the cells. Three surrounding ROIs were also selected for each DRG soma, to provide background intensity over time. The fluorescence changes ΔF/F_B_, is defined as (Ft-F_B_/F_B_)%, where Ft is the fluorescence intensity at time t, while F_B_, is the average 6 seconds fluorescence intensities, 28 seconds before the stimulation. The same cells were used for calcium measurements in Configurations D and C. Time lapse imaging was carried out every 2 seconds. Only increase of 10% or more was considered an event, for each imaging series. The frequencies of each ΔF/F_B_ event were analyzed in two groups; 28 seconds before stimulations and 154 seconds during stimulation. The results were expressed as mean ± SEM (n = 10 cells). Statistical analysis was performed with paired student-t test (α = 0.05).

### Immunostaining and imaging

Co-cultures were fixed with 4% paraformaldehyde by incubation for 30 min at room temperature and subsequent rinsing with Dulbecco’s phosphate buffered saline (DPBS, Gibco). Cells were treated for 30 min in room temperature with a blocking buffer containing 5% goat serum (Sigma) and 0.1% Triton (Sigma) in DPBS. Subsequently samples were incubated with primary antibodies diluted in blocking buffer overnight at 4°C. Primary antibodies included: mouse anti-O4 (Millipore, 1:500), mouse anti-myelin basic protein (MBP; Millipore, 1:1000), mouse or rabbit anti-neurofilament (Cell Signaling, 1:500), and rabbit anti-CNPase (Cell Signaling, 1:1000). Cells were washed twice with DPBS and incubated with two of the following secondary antibodies diluted in blocking buffer for 30 min at room temperature (RT): Alexa488 goat anti-mouse (1:200; Life Technologies), Alexa488 anti-rabbit (1:200; Molecular Probes), Alexa594 goat anti-rabbit (1:200; Life Technologies), Alexa594 anti-mouse(1:200; Molecular Probes). Finally, cells were washed with DPBS and deionized water, and mounted in antifade solution (prolong gold antifade; Life Technologies). Imaging was performed using an inverted, confocal microscope (Zeiss). Only the area of the axonal compartment that contained axons was imaged. The numbers of cell with high O4, MBP or CNPase signals within that area were counted manually. We only considered OPCs as O4-positive if they had a high immunoreactivity signal. To achieve a standardized threshold, the gain of the confocal microscope was set at a level to remove the low-gain O4 signal. The myelin fragments with MBP and neurofilament signal colocalization were counted manually. Each experimental group contained at least 10 independent experimental replicates performed on cell cultures from at least 3 independent animal dissections.

### Statistical analysis

Unless indicated otherwise, the statistical comparisons were performed by one-way ANOVA with Bonferroni-Holm post-hoc testing with statistical significance at α = 0.05.

## Results and discussion

### Whole cell and subcellular stimulation with electric field simulations

We aimed to achieve subcellular electrical stimulation (ESTIM) by exploiting the compartmentalized neuron/glia co-culture. To understand the characteristics of the electric field in the compartmentalized system, we performed a set of simulations for different sizes of compartments and different configurations of the platinum electrodes. Our microfluidic platform contained two compartments, somatic and axonal, connected with 2.5-μm-high, 10-μm-wide, and 500-μm-long microchannels. The compartments were open to the air to provide an access, ease of manipulation, and buffering capacity. The dimensions of the compartments were: 7 mm length, 4 mm width and 7 mm height. The volume of each compartment was approximately 200 μL to limit evaporation and provide sufficient media for cell culture.

Our compartmentalized culture system was integrated with electrical stimulation (Fig 1A and 1B). To better understand the electric field characteristics, we perform a set of finite element simulations for different configuration of electrodes (see Materials and methods). When the electrodes were placed in the mid-width of each compartment the field magnitude in the middle between the electrodes was 20.3% of the maximum observed at the electrodes (Fig 1C and 1E). 47.72% of the compartments surface area was exposed to the effective field, defined as the region with electric field magnitude within 10% of the maximum. When the electrodes were shifted to the top of the device (Fig 1D) the effective field covered only 28.89% of the compartments.

We then simulated the electric field when the electrodes were positioned diagonally within axonal compartment (Fig 1E) and in two different compartments (Fig 1F). The first configuration generated electric fields predominantly in the axonal compartment; however, 9.48% of the somatic compartment was exposed to the effective field (Fig 1J). Placing the electrodes in the diagonal corners of two different compartments limited the magnitude of the field between them to 6.12% of the maximum and only 23.03% area of the compartments was covered by the effective field. Finally, we simulated the electric field when the two electrodes were located 4 mm apart in the middle of the somatic (Fig 1G) or axonal (Fig 1H) compartments. In both scenarios, the effective field was fully restricted to the compartment where the electrodes were placed and covered 93.54% of the area of its area. The field magnitude in the middle between the electrodes was 22.76% of the maximum (Fig 1I). To verify simulation results, we measured the electric potentials at four central locations for the WholeSTIM paradigm (Fig 1C) and two central locations for the focal paradigm (Fig 1E). In each configuration, the electric field was estimated by normalizing the voltage measured across each pair of electrodes to the separation length (Fig 1C–1H), enabling direct comparison to the electric field amplitude obtained through simulation (Fig 1L). The measurements show that the relative amplitudes between the measurement electrodes are in good agreement with the electric field distributions obtained through simulations.

Simulations and measurements reveal that electrode localization is a key parameter in performing selective stimulation within a compartmentalized culture system. As neurons require stimulation above thresholds to induce activity [27], irregularity and spatial restriction of the electric field has several practical consequences for experimental design: (1) placement of the electrodes in the middle of the compartment achieves the highest spatial coverage for simultaneous exposure of somatic and axonal compartments; (2) localization of electrodes in the far corners of the two compartments provides the least coverage of the two compartments; (3) placement of electrodes in the middle of the compartment can restrict the field to a single compartment, according to simulations; (4) diagonal placement of the electrodes even within the same compartment can result in above threshold electric fields in the opposite compartment, according to the simulations.

Intrigued by the spatial restriction of the electric field in our system, we performed another set of simulations to test how the dimensions of the compartments affect the coverage with the effective electric field. This information is important considering that commercially available compartmentalized systems for neuron culture can have a wide range of compartments dimensions. The length of somatic and axonal compartments in our simulation was increased to 15 mm (Fig 2A and 2B) and three electrode placements were considered for simultaneous stimulation of both compartments. The mid-length and mid-width electrode configuration (Fig 2C) provided effective field coverage of only 19.95% of the compartments surface area. The minimum field magnitude between the electrodes was 14.54% of the maximum. Moving the electrodes diagonally (Fig 2D) increased the effective field coverage to 22.82% but decreased the minimum field magnitude between the electrodes to 8.11% of the maximum. Finally, placing the electrodes in the far corners of the device (Fig 2E) minimized the compartments surface area exposed to the effective field to 5.91% and decreased the minimum field magnitude between the electrodes to 2.49%. These results further emphasize the need for careful consideration of the electrical stimulation system, especially when using commercially available neuron compartmentalized cultures.

Compartmentalized culture integrated with the platinum rods as electrodes is an easy and versatile system for electrical stimulation [28]. If the homogeneity of the electric field is a crucial element of the experimental design other methods should be considered, e.g., microelectrode arrays for electrical stimulation [29].

### Integrated compartmentalized co-culture system with subcellular ESTIM

Dorsal root ganglion neurons (DRGs) were plated in the somatic compartment of the bicompartmental device. Cell bodies extended neurites, which extensively elongated and by 4 day *in vitro* (DIV) crossed to the axonal compartment. At 10 DIV high number of axonal processes was present in the axonal compartment and this is when oligodendrocyte precursor cells (OPCs) were plated in that compartment. The set of parallel microchannels connecting somatic and axonal compartment allowed axons, but prohibited cell bodies from passing, providing spatial isolation of neuronal cell bodies and distal axons with OPCs. Accordingly, no OPCs were observed in the somatic compartment, confirming the spatial isolation and exclusive contact between OPCs and distal axons. Good viability of the cells was sustained for at least 14 days in co-culture. After 3 days OPCs started developing processes typical for oligodendrocytes (OLs) indicating their differentiation. In comparison to cortical and hippocampal neurons, DRGs in *in vitro* culture demonstrate relatively little spontaneous electric activity and synchronization. This feature makes it easier to distinguish the effects of externally induced neuron activity over the effects of the inherent firing.

**Fig 3:**
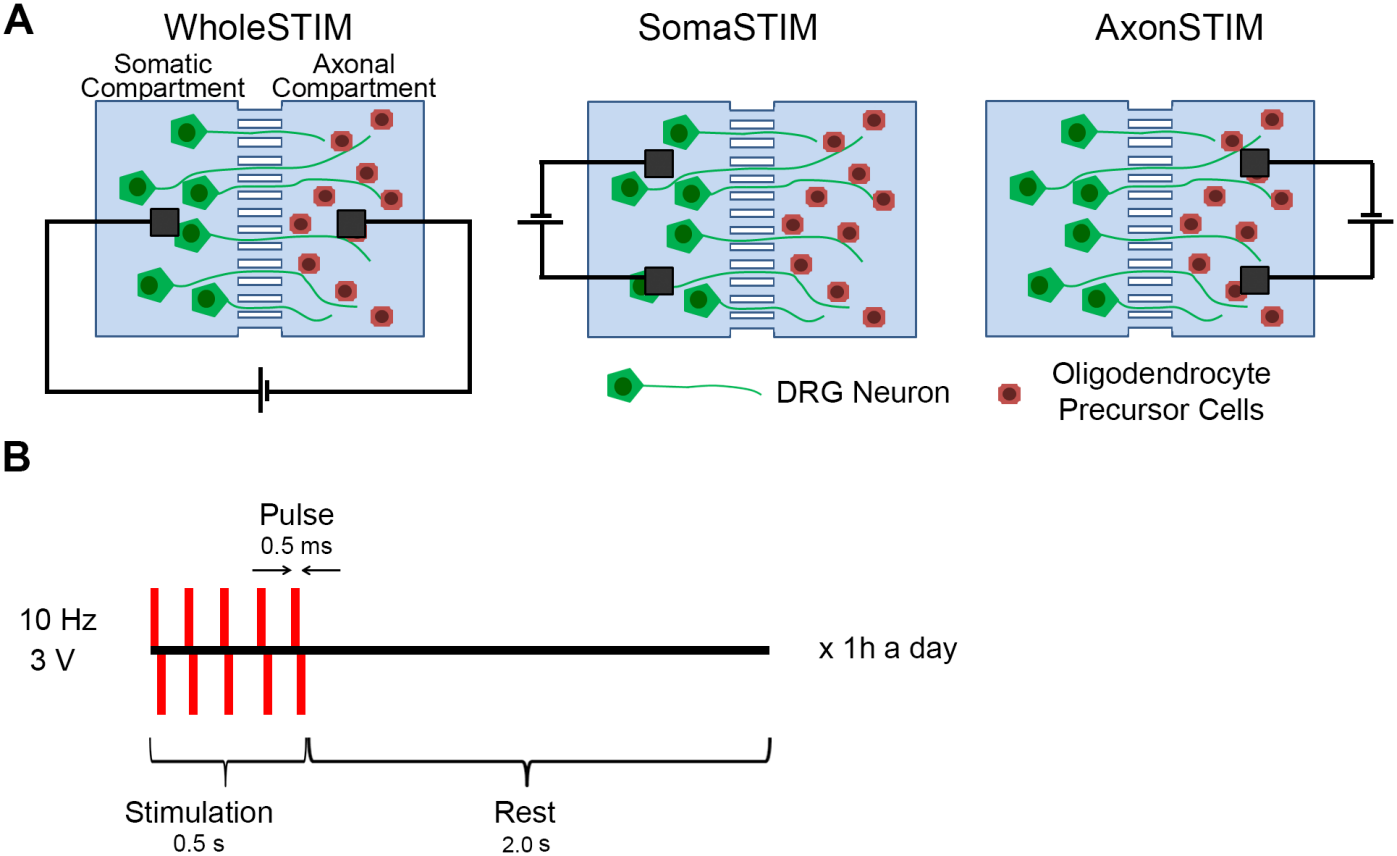
Integrated compartmentalized and electrical system for subcellular stimulation of neurons. (A) Dorsal root ganglion neurons and oligodendrocyte precursor cells were cultured in microfluidics and electrically stimulated in three different modes of electrode configuration: whole cell stimulation (WholeSTIM), somatic compartment stimulation (SomaSTIM) and axonal compartment stimulation (AxonSTIM). (B) Schematic of stimulation protocol.

ESTIM was performed for 1h a day in DRG/OPC co-cultures starting on the day of OPCs plating. Three configurations of electrodes were used to induce neuronal activity: (1) WholeSTIM (Fig 1C): This configuration stimulated the cell bodies in the somatic compartment and distal axons with OPCs in the axonal compartment; (2) SomaSTIM (Fig 1G): This configuration induced neuronal activity of the cell bodies and proximal axons, while the myelination was assessed in the axonal compartment; (3) AxonSTIM (Fig 1H): This configuration stimulated the distal axons in the axonal compartment as well as oligodendrocytes (Fig 3A). The cells were stimulated with 10 Hz, 3 V-high, 0.5 ms-wide rectangular biphasic pulses forming trains that last for 0.5 s, repeated after 2 s of rest for 1 hour a day[13]. This stimulation regime is in the range of physiological firing rate of the DRGs at later stages of development (1–10 Hz), and following target stimulation (10–20 Hz)[30]. The stimulation parameters were the same for all the ESTIM modes and were previously used to study activity-dependent myelination (Fig 3B) [13, 18, 25]. The control culture contained electrodes not connected to the power source.

### ESTIM effects on axon activation

**Fig 4:**
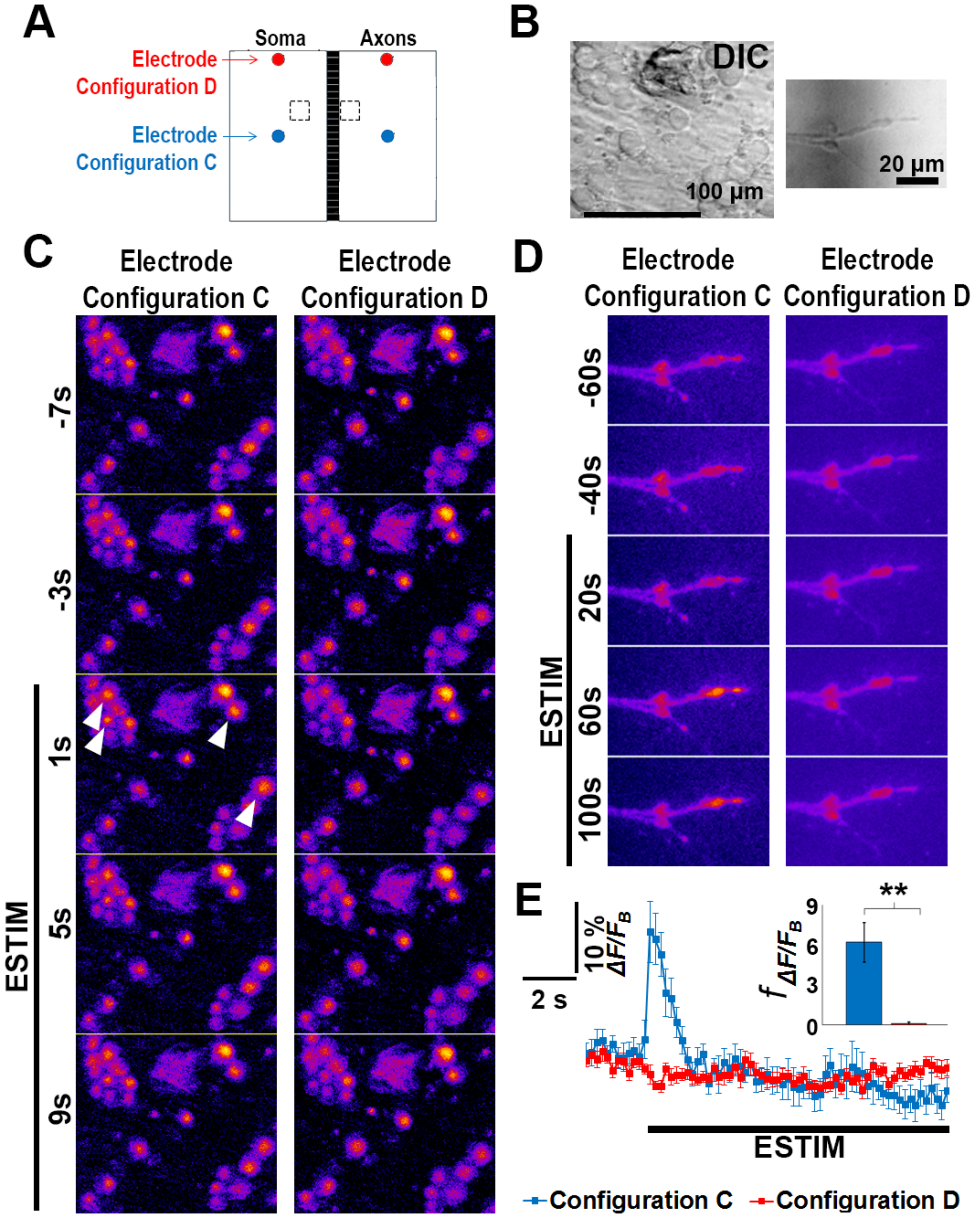
Electrode configuration affects the effectiveness of neuron stimulation. (A) The same central regions of a microfluidic neuron culture (broken line boxes) were first imaged under electrode configuration D than electrode configuration C. (B) DIC images of the representative region of the imaged areas. (C-D) Calcium response of cell bodies (*C*) and axons (*D*) to ESTIM. E Average calcium changes (ΔF/F_B_) pooled from the same cell bodies in two different electrode configurations. The inset shows frequency of the firing events (f_ΔF/FB_) in 3 min following ESTIM onset. The results are expressed as mean ± SEM (n = 10 cells). Statistical analysis was performed with paired student-t test ** p<0.005.

cAMP level has been shown to increase in response to neuron activation and to consequently mediate axon myelination [13]. We measured cAMP in our system to confirm its capability to activate neurons. Indeed, when the cells were stimulated in a WholeSTIM paradigm for 1h and cAMP level was measured 1 h later *via* a competition enzyme-linked immunoassay we observed a 37.5% increase in cAMP concentration compared to the unstimulated control (S3 Fig and S1 File). To test the spatial distribution of neuron activation after ESTIM depending on the electrodes’ positions we measured calcium activity (Fig 4). The same neurons in the central locations of the culture (Fig 4A) were imaged for two different electrodes configuration, C and D, and their calcium levels were analyzed before and during ESTIM (Fig 2A and 2B). Configuration D failed to evoke neuron firing in axons and cell bodies (Fig 2C–2E). Configuration C evoked the calcium elevation in axons as well as synchronized firing in the responsive cell bodies upon ESTIM onset as indicated by increased calcium level (ΔF/F_B_) and elevated firing events frequency (*f*_ΔF/FB_) (Fig 4E). ΔF/F_B_ traces for individual cells are shown in S4 Fig.

### Subcellular specific ESTIM promoted the differentiation of oligodendrocytes and myelination

**Fig 5:**
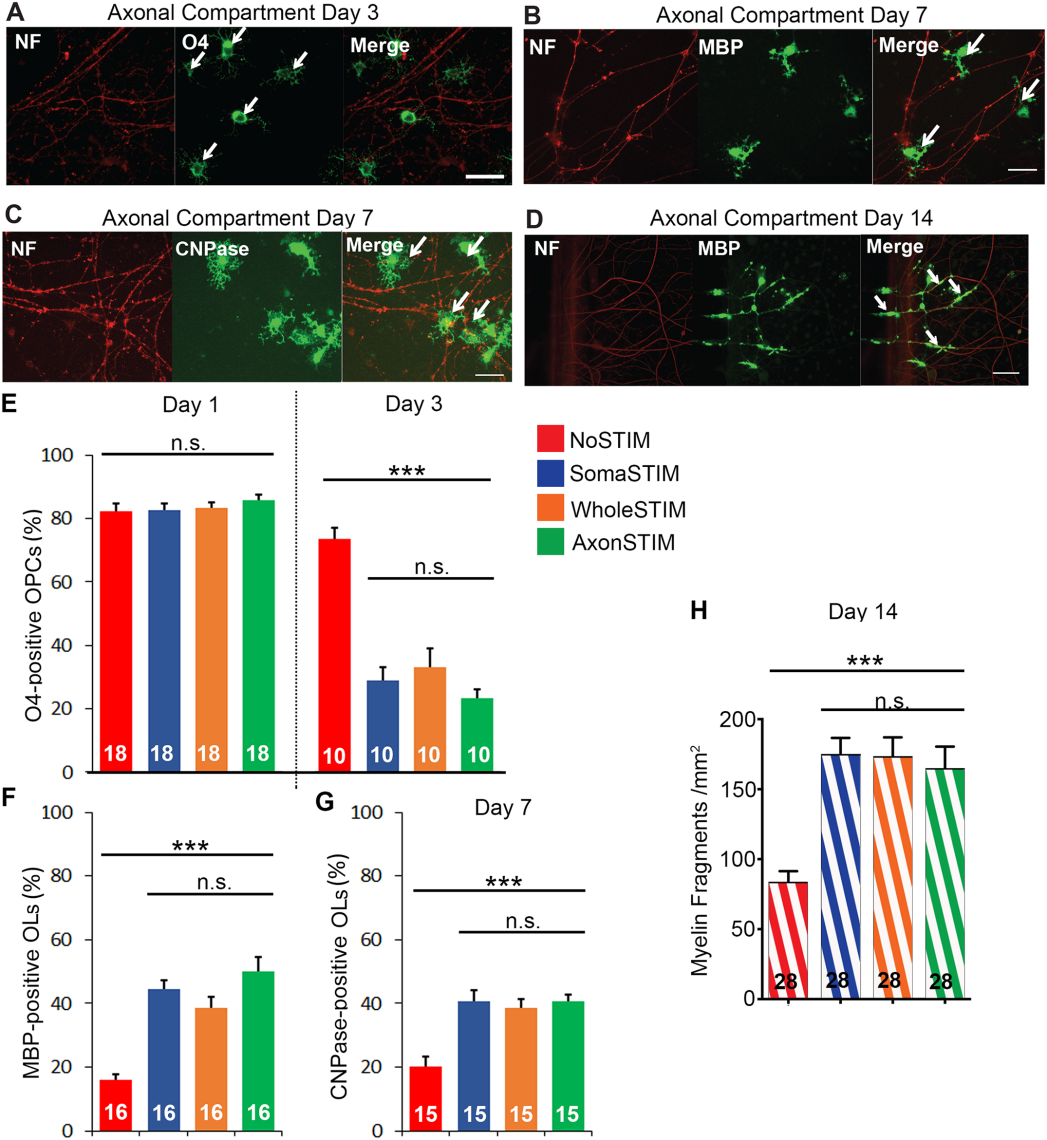
Whole cell and subcellular ESTIM enhances oligodendrocyte precursor cells (OPCs) differentiation into oligodendrocytes (OLs) and supports axon myelination. (A-D) Representative images of axons in the axonal compartment stained against neurofilaments (NF, red) and OLs stained against a set of differentiation markers: (A) O4 signal (green) expressed by premature OPCs (white arrows) after 3 days of stimulation; (B) CNPase signal (green) expressed by mature OLs (white arrows) after 7 days of stimulation; (C) MBP signal (green) expressed by mature OLs (white arrows) after 7 days of stimulation; (D) MBP signal (green) expressed by mature OLs after 14 day of stimulation demonstrating formed myelin fragments (white arrows). Scale bar (A—D): 50μm. The images for all experimental conditions are shown in S6 Fig. (E) The percentage of O4-positive OPCs decreased slightly after 3 days in the control group outlining the baseline level of the differentiation. ESTIM further decreased the percentage of O4-positive OPCs. (F-G) 7 days of ESTIM supported OLs maturation as indicated by the increase in the percentage of (F) MBP-positive and (G) CNPase-positive cells. (H) 14 days of ESTIM increased the number of formed myelin fragments. The data are presented as mean ± S.E.M. For individual data points see S7 Fig. The numbers on the graphs stand for the numbers of experimental replicates. The groups were compared with one-way ANOVA at a significance level of α = 0.05; *** p<0.001.

The OPCs were plated in the axonal compartment and co-cultured with axons for 14 days. The viability of the OPCs decreased significantly in the first week of the culture, but was stable in the later stage when the cultures were more established (DIV 7 –DIV 14). ESTIM did not affect the cell number compared to control (S5 Fig). To quantify the effects of ESTIM on axon myelination, we tested if it enhanced OPCs differentiation into OLs that have a capability to wrap around the axons. We applied ESTIM in all three configurations, fixed the cells and stained for O4 –marker of the premature OPCs [31]. Although the O4 expression is maintained throughout the OPCs differentiation towards OLs, its expression is reduced with the maturation of OPCs [32]. We only considered OPCs as O4-positive if they had a high immunoreactivity signal. 1 day of ESTIM did not affect the percentage of premature OPCs as visualized with anti-O4 antibody (Fig 5A and 5E). However, after 3 days of ESTIM in all three electrode configuration, we observed a substantial reduction of the premature OPCs population as demonstrated by a decrease of O4-positive OPCs. This decline was significantly more pronounced than the modest decrease (statistically significant, t-test, p<0.01) observed in the control culture. These results suggest that the externally evoked neuron activity was not necessary for OPCs differentiation, but significantly enhanced it. Interestingly, no differences were detected in the results of ESTIM in three different electrodes configurations.

We then aimed to test if the decrease in the number of premature OPCs was followed by the increase in the number of mature OLs. We fixed the cells after 7 days of the stimulation and stained glia cells against myelin basic protein (MBP) and 2',3'-Cyclic-nucleotide 3'-phosphodiesterase (CNPase)–the markers of mature OLs [31] (Fig 5B and 5C). The percentages of MBP-positive and CNPase-positive cells were significantly higher after ESTIM than in the control cultures (Fig 5F and 5G). Consistent with O4 staining results, no differences were detected in the effects of the electrode configurations.

Subsequently, we aimed to test if the increased differentiation of OPCs into OLs resulted in increased myelination. The full overlapping of neuronal processes with MBP-positive OLs structures has been shown before to indicate a formation of early myelin fragments [13, 17], therefore, we stimulated the co-cultures for 14 days, fixed and stained them against MBP (Fig 5D). The number of the early myelin fragments in the unstimulated control culture was 78 / mm^2^. This number is much higher than previously published results when the compartmentalized neuron/OPCs co-cultures were used [13, 19]. This is caused by restricting the quantification to the areas covered by axons and using a higher seeding density of the OPCs. We observed that ESTIM significantly increased the number of the early myelin fragments independent of its mode. Taken together, these data strongly imply that ESTIM enhanced differentiation of premature, O4-positive OPCs into MBP-positive and CNPase-positive mature OLs and accordingly, increased the number of early myelination fragments.

The increase in the OLs maturation and early myelin formation after whole cell stimulation (WholeSTIM) is consistent with those reported before [13, 18, 19]. Our results demonstrate that subcellular stimulation is sufficient to induce activity-dependent axon myelination. Stimulation of the somatic compartment (SomaSTIM) activated cell bodies and proximal axons and resulted in the anterograde signal propagation to the distal axons in the axonal compartment that were not directly stimulated. This stimulation was sufficient to induce the same effect as the whole cell stimulation. Similarly, when the electrodes were located in the axonal compartment (AxonSTIM), we observed an increase of the OPCs differentiation and early myelin formation on the same level as after WholeSTIM and SomaSTIM. Two plausible mechanisms can explain this observation: (1) activated axons propagated signal to the cell bodies and evoked global response; (2) local axonal activity induction is sufficient to induce activity-dependent myelination. Furthermore, in this mode, both distal axons and oligodendrocytes were exposed to the electric field. Recent studies show that OPCs can sense and migrate in the stable, small electric fields [33, 34]. Therefore, it cannot be excluded that OPCs directly responded to the stimulation. Yet, we did not observe an increased myelination after WholeSTIM and AxonSTIM (co-treatment of neurons and OPCs) when compared to SomaSTIM (stimulation of neurons only), suggesting that axon myelination in response to the electric field stimulation is predominantly driven by neuronal activity. Although these questions are out of the scope of this study, our experimental platform offers multiple advantageous for further investigations.

The effects of ESTIM are in agreement with the recent reports from *in vivo* studies showing that neural activity supports and stabilizes myelin sheath formation by oligodendrocytes [12, 14, 35]. As the induction of signal activity in a brain *in vivo* usually does not facilitate classification of the stimulation to whole cell, axons or soma, it is plausible to assume that the observed effects on myelination are an outcome of all those modes of treatment. Therefore, it is important to understand if there are differences in the effects of the focal *vs*. global stimulation and between stimulation of neurons only *vs*. co-stimulation of neurons and OPCs. Our input restriction assay facilitated testing this idea and demonstrated that focal axonal and somatic stimulations are sufficient to evoke OPCs differentiation into OLs and early myelin formation. These results not only provide mechanistic insight into the activity-dependent myelination but also may be useful for designing therapeutic strategies for de-myelination diseases.

## Conclusions

It has become abundantly clear that the role of myelination in the central nervous system expands beyond expediting electrical signal propagation. One of the key challenges in the emerging field of myelin plasticity is to link changes in neuron activity with specific myelination profiles [2]. Answering this and other questions require new tools. In this study, we present a compartmentalized neuron/oligodendrocyte co-culture device integrated with electrical stimulation system that allows applying whole cell and spatially restricted cell activity induction. This platform offers multiple advantages for studying activity-induced myelination, including: (1) ease of manipulation and integration with standard optical and molecular biology techniques; (2) restriction of oligodendrocyte interaction to distal axons to better replicate the *in vivo* scenario; and finally, (3) spatial restriction of the electrical stimulation input to a chosen subcellular region of neurons. There is no surprise that the popularity of the compartmentalized culture systems for neuronal studies has been increasing for the last decade. Commercially available systems can be easily integrated with the electrical stimulation. However, our simulations of the electric field emphasize that electrode locations have to be carefully designed. Using too sparsely located electrodes in somatic and axonal compartment may result in an uneven electric field, and cause three different spatial modes of neuron activity induction (whole cell, somatic and axonal) within one culture. Finally, we demonstrate that restricting electrical stimulation to distal axons with OPCs or neuronal cell bodies is sufficient to induce activity-dependent axon myelination on the same level as whole cell electrical stimulation. This intriguing observation has to be further investigated.

## Supporting information

S1 Fig Oligodendrocyte precursor cells (OPCs) viability after plating. OPCs purified *via* magnetic-activated cell sorting (MACS) had a high viability as demonstrated by their morphology after plating. The cells attached to the glass substrate and started extending processes on DIV 1. Extensive branching of the processes was observed on DIV 7. DIV, day *in vitro*.
(TIF)

S2 Fig Individual data points for electric potential measurement in Fig 1. The results for electrode configuration G and H are obtained from the same set of measurements.
(TIF)

S3 Fig cAMP measurement. Whole cell electrical stimulation (ESTIM) increased cAMP level detected in cell lysate compared to the unstimulated control (NoSTIM). Values are mean ± S.E.M. Diamond markers show individual data points. *p<0.05 compared by unpaired student t-test at α = 0.05.
(TIF)

S4 Fig Individual calcium traces of neurons under ESTIM in the peripheral (D) and central (C) electrode configurations shown in Fig 4. Calcium level changes (ΔF) were normalized to the baseline level (calcium level prior ESTIM; F_B_). Most of the neurons fired in a synchronized manner at the time of ESTIM application (t = 0 s) for electrode configuration C (blue), but not in configuration D (red).
(TIF)

S5 Fig OPC number does not depend on ESTIM. Cell density (number of cells per mm^2^) as a function of time. Data points represent experimental replicates. Cell number was determined by DAPI counterstaining. Differentiation marker-positive cells are counted within the same images (Fig 5). The data for DIV 7 were pulled together from the data sets for immunostaining of CNPase and MBP. The data are compared with ANOVA at α = 0.05; ***p<0.001. DIV, day *in vitro*.
(TIF)

S6 Fig OPCs differentiation markers at Day 1 to Day 14 in the control and ESTIM groups. After the stimulation the cells were fixed, and stained with DAPI (blue) and antibody against a differentiation marker (green). O4—the marker of premature OPCs—was visualized at Day 1 and Day 3. 2',3'-Cyclic-nucleotide 3'-phosphodiesterase (CNPase) or myelin base protein (MBP)—the markers of oligodendrocytes—were visualized at Day 7. MBP staining at Day 14 was used to visualize myelin fragments forming around axons (red, stained with anti-neurofilament antibody). Scale bar = 50 μm.
(TIF)

S7 Fig Individual data points for ESTIM effects on OPCs differentiation and axon myelination shown in Fig 5. Data points represent individual experimental replicates for the quantification of marker-positive cells and myelin fragments.
(TIF)

S1 File Methods: cAMP level measurement. (PDF)
