# Supplementary figures and images for: Subcellular electrical stimulation of neurons enhances the myelination of axons by oligodendrocytes
Source: PLoS One. 2017 Jul 3;12(7):e0179642. doi: 10.1371/journal.pone.0179642 (PMC5495216; doi:10.1371/journal.pone.0179642)

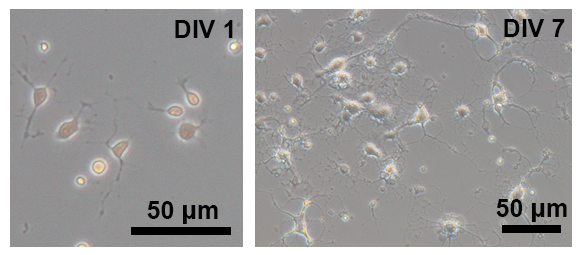

Supplement: S1 Fig — OPCs purified via magnetic-activated cell sorting (MACS) had a high viability as demonstrated by their morphology after plating. The cells attached to the glass substrate and started extending processes on DIV 1. Extensive branching of the processes was observed on DIV 7. DIV, day in vitro. (TIF) [file pone.0179642.s001.tif]

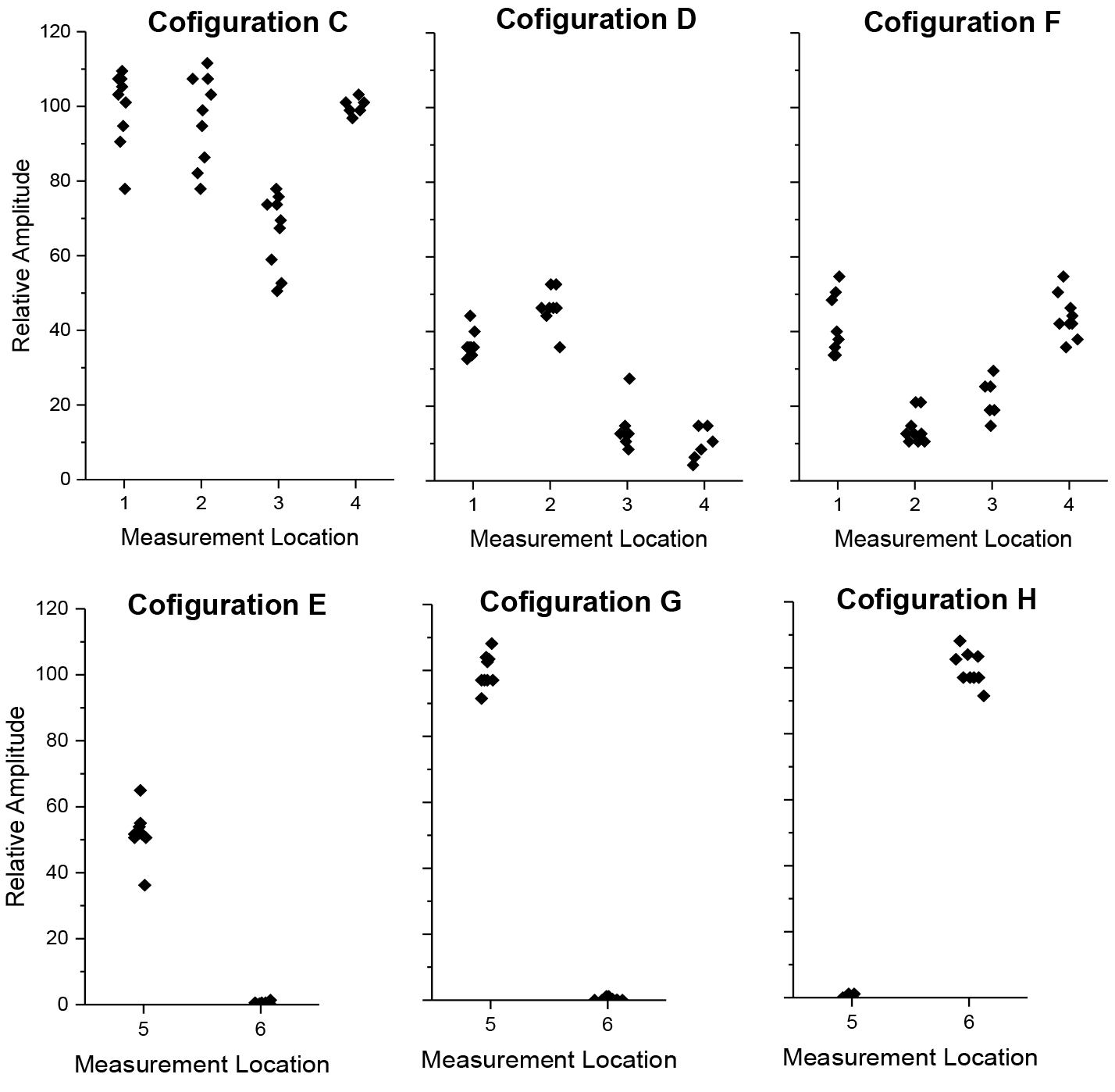

Supplement: S2 Fig — The results for electrode configuration G and H are obtained from the same set of measurements. (TIF) [file pone.0179642.s002.tif]

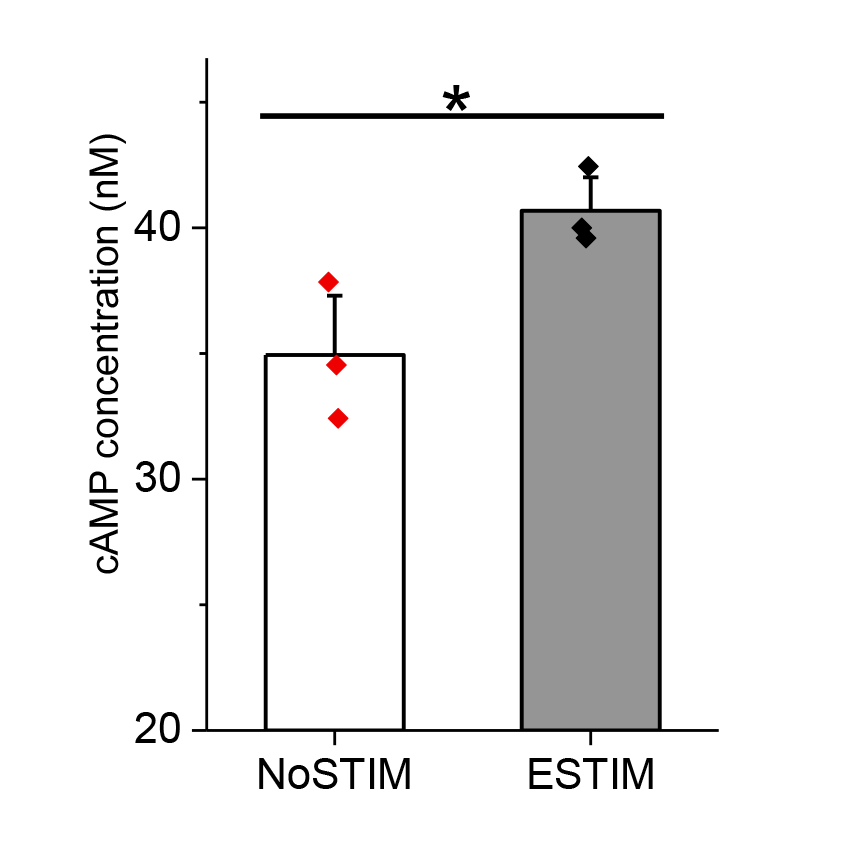

Supplement: S3 Fig — Whole cell electrical stimulation (ESTIM) increased cAMP level detected in cell lysate compared to the unstimulated control (NoSTIM). Values are mean ± S.E.M. Diamond markers show individual data points. *p<0.05 compared by unpaired student t-test at α = 0.05. (TIF) [file pone.0179642.s003.tif]

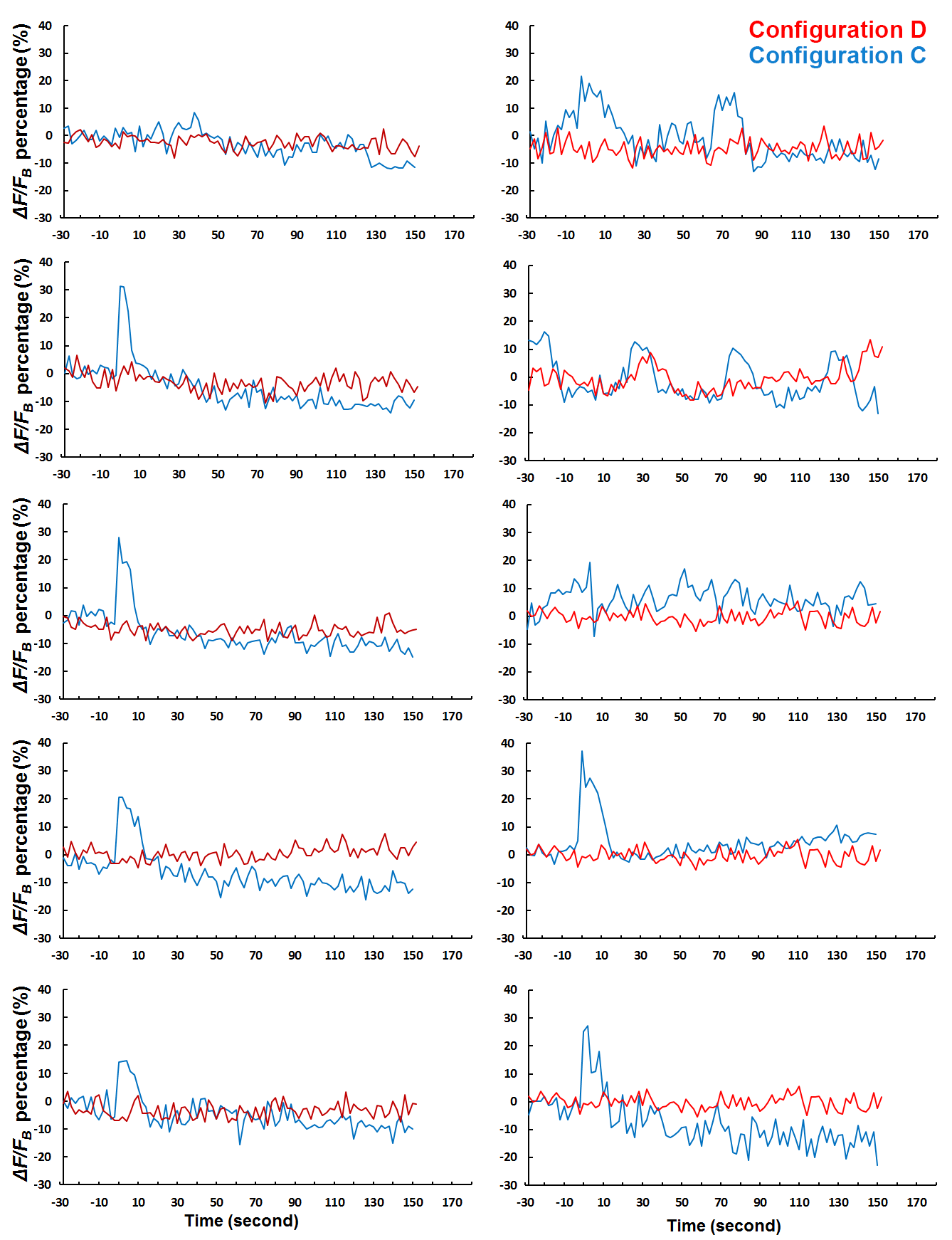

Supplement: S4 Fig — Calcium level changes (ΔF) were normalized to the baseline level (calcium level prior ESTIM; FB). Most of the neurons fired in a synchronized manner at the time of ESTIM application (t = 0 s) for electrode configuration C (blue), but not in configuration D (red). (TIF) [file pone.0179642.s004.tif]

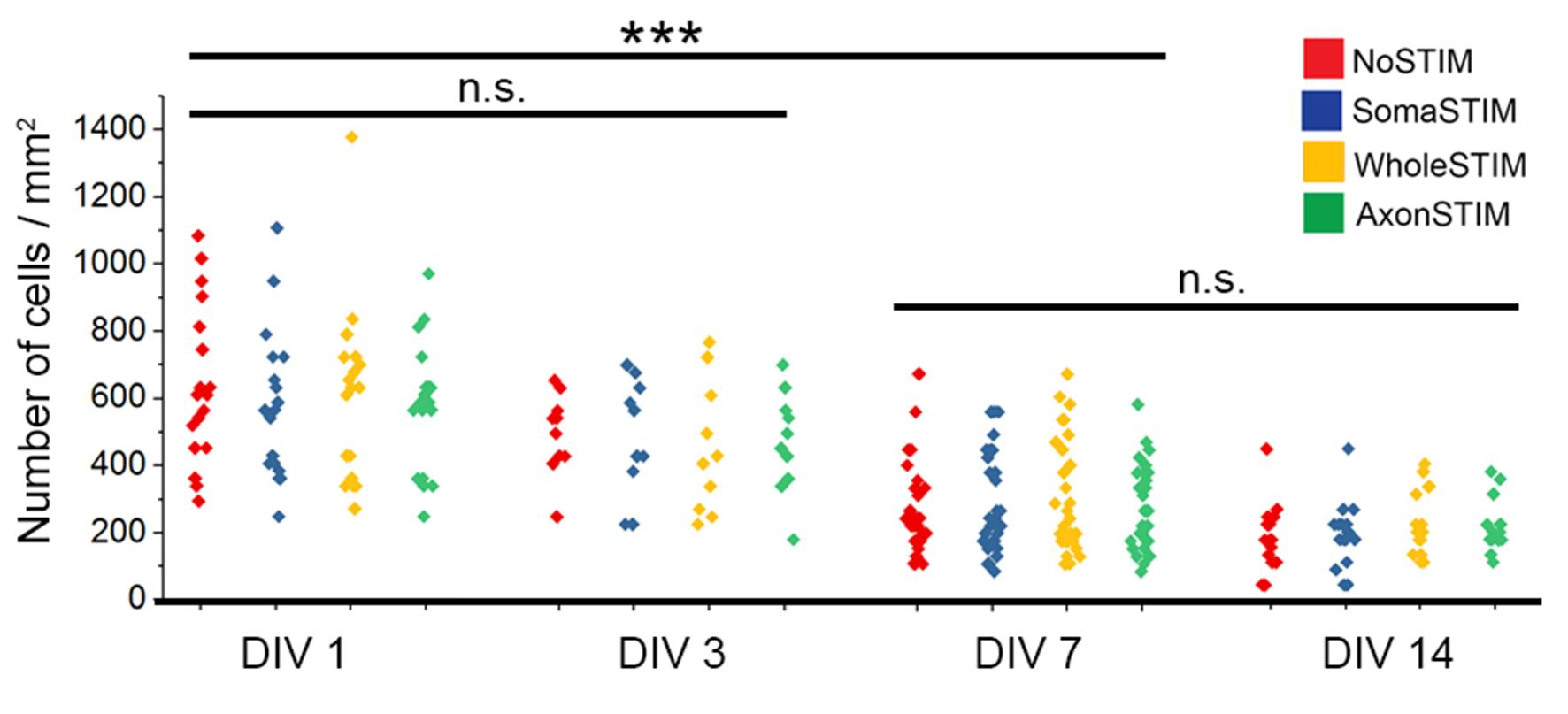

Supplement: S5 Fig — Cell density (number of cells per mm2) as a function of time. Data points represent experimental replicates. Cell number was determined by DAPI counterstaining. Differentiation marker-positive cells are counted within the same images (Fig 5). The data for DIV 7 were pulled together from the data sets for immunostaining of CNPase and MBP. The data are compared with ANOVA at α = 0.05; ***p<0.001. DIV, day in vitro. (TIF) [file pone.0179642.s005.tif]

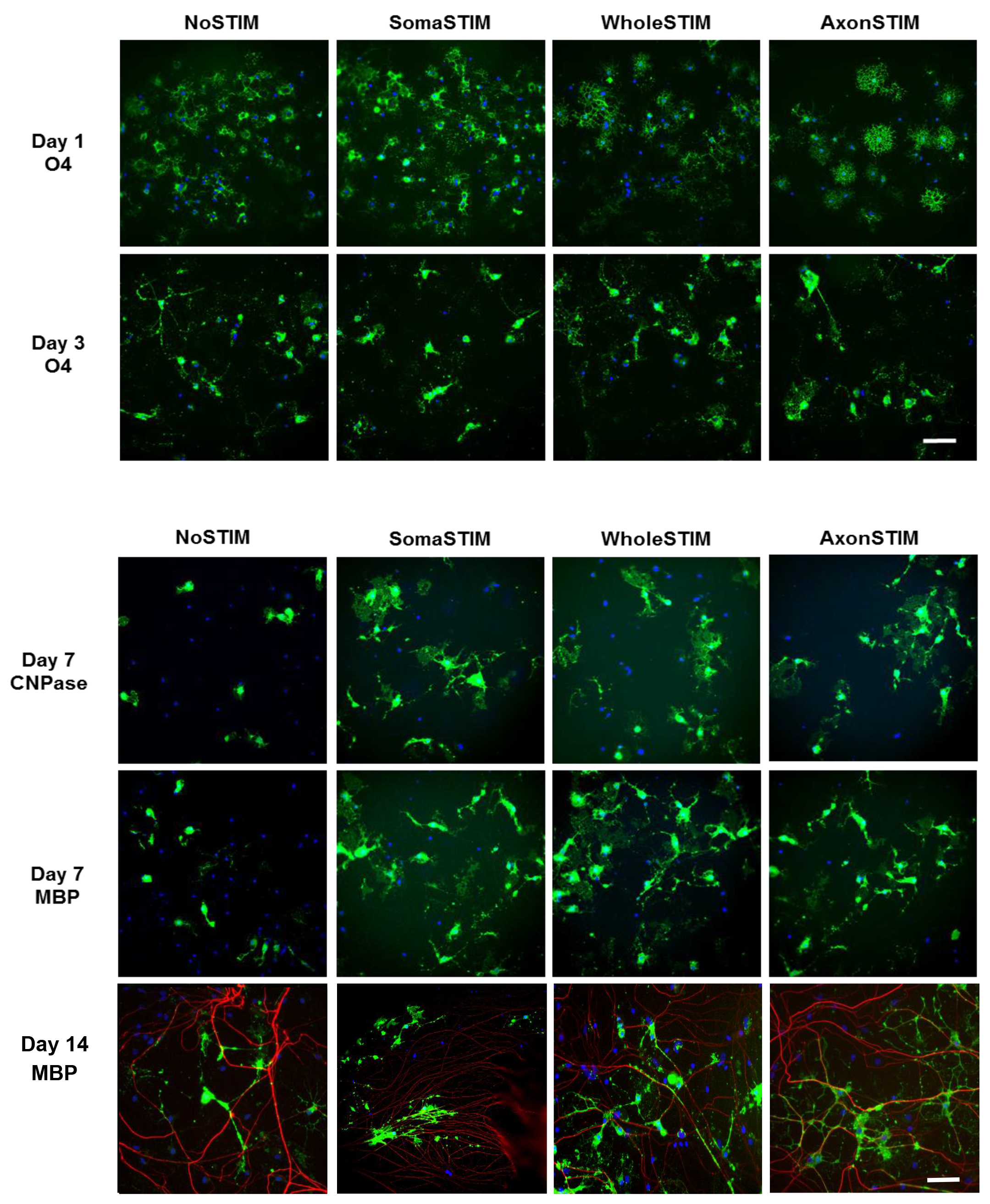

Supplement: S6 Fig — After the stimulation the cells were fixed, and stained with DAPI (blue) and antibody against a differentiation marker (green). O4—the marker of premature OPCs—was visualized at Day 1 and Day 3. 2',3'-Cyclic-nucleotide 3'-phosphodiesterase (CNPase) or myelin base protein (MBP)—the markers of oligodendrocytes—were visualized at Day 7. MBP staining at Day 14 was used to visualize myelin fragments forming around axons (red, stained with anti-neurofilament antibody). Scale bar = 50 μm. (TIF) [file pone.0179642.s006.tif]

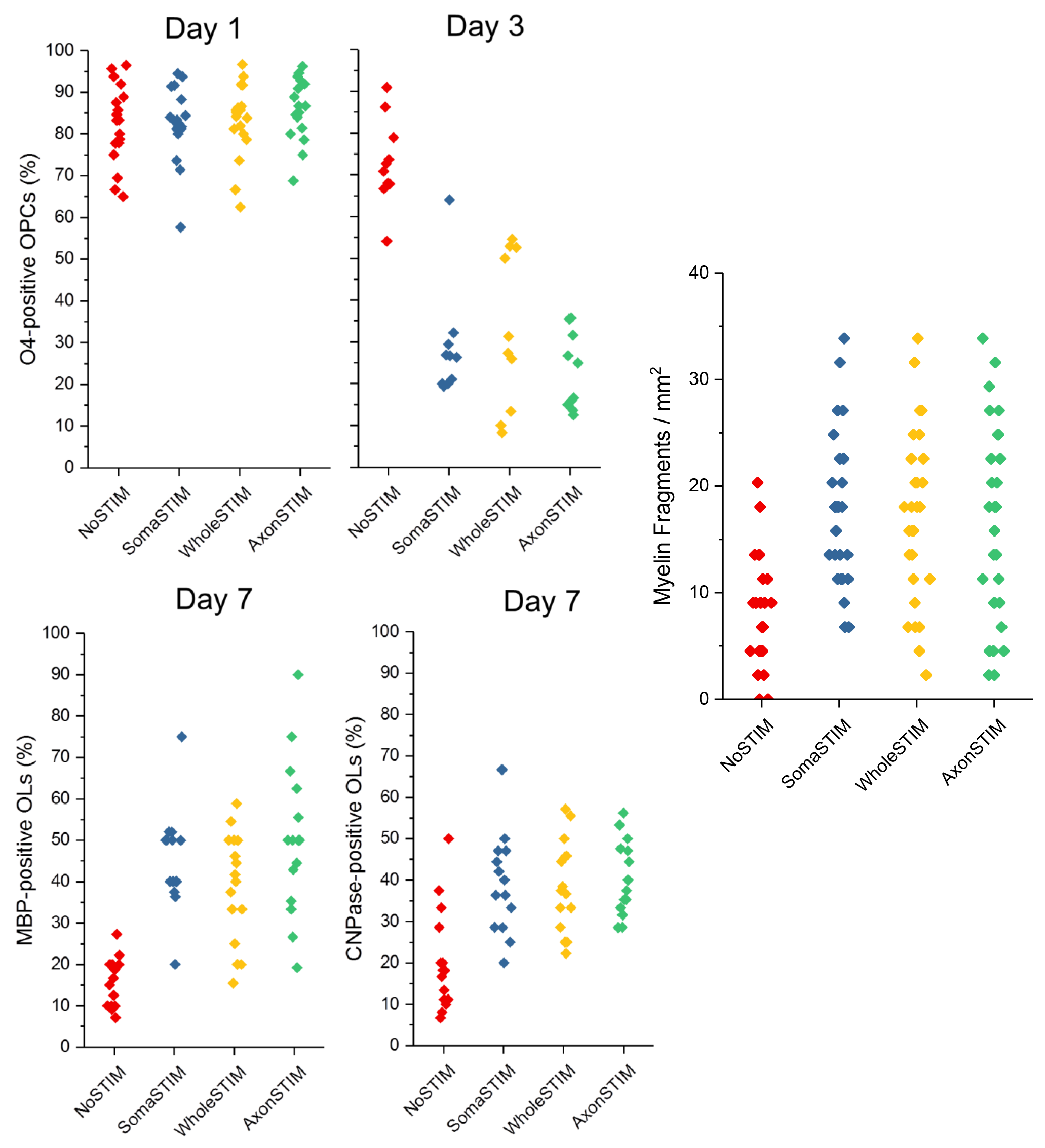

Supplement: S7 Fig — Data points represent individual experimental replicates for the quantification of marker-positive cells and myelin fragments. (TIF) [file pone.0179642.s007.tif]
