# Supplementary material for: Subcellular electrical stimulation of neurons enhances the myelination of axons by oligodendrocytes
Source: PLoS One. 2017 Jul 3;12(7):e0179642. doi: 10.1371/journal.pone.0179642 (PMC5495216; doi:10.1371/journal.pone.0179642)
Supplement: S1 File — (PDF) [file pone.0179642.s008.pdf]

S1 File. Methods: cAMP Level Measurement.

Measurement was performed on neurons 1 hour after ESTIM. Neurons were rinsed with PBS and incubated for 10 min with 1 mM phenylmethylsulfonyl fluoride (PMSF, Cell Signaling) in lysis buffer (Cell Signaling). The cell lysate was then analyzed with Cyclic AMP XP® Assay Kit (Cell Signaling) following manufacturer's instructions.
